# A Robust Tri-Electromagnet-Based 6-DoF Pose Tracking System Using an Error-State Kalman Filter

**DOI:** 10.3390/s24185956

**Published:** 2024-09-13

**Authors:** Shuda Dong, Heng Wang

**Affiliations:** Shien-Ming Wu School of Intelligent Engineering, South China University of Technology, Guangzhou 511442, China; wishudadong@mail.scut.edu.cn

**Keywords:** pose estimation, electromagnetic tracking, localization, magnetic sensor, Kalman filter

## Abstract

Magnetic pose tracking is a non-contact, accurate, and occlusion-free method that has been increasingly employed to track intra-corporeal medical devices such as endoscopes in computer-assisted medical interventions. In magnetic pose-tracking systems, a nonlinear estimation algorithm is needed to recover the pose information from magnetic measurements. In existing pose estimation algorithms such as the extended Kalman filter (EKF), the 3-DoF orientation in the $S^{3}$ manifold is normally parametrized as unit quaternions and simply treated as a vector in the Euclidean space, which causes a violation of the unity constraint of quaternions and reduces pose tracking accuracy. In this paper, a pose estimation algorithm based on the error-state Kalman filter (ESKF) is proposed to improve the accuracy and robustness of electromagnetic tracking systems. The proposed system consists of three electromagnetic coils for magnetic field generation and a tri-axial magnetic sensor attached to the target object for field measurement. A strategy of sequential coil excitation is developed to separate the magnetic fields from different coils and reject magnetic disturbances. Simulation and experiments are conducted to evaluate the pose tracking performance of the proposed ESKF algorithm, which is also compared with standard EKF and constrained EKF. It is shown that the ESKF can effectively maintain the quaternion unity and thus achieve a better tracking accuracy, i.e., a Euclidean position error of 2.23 mm and an average orientation angle error of 0.45°. The disturbance rejection performance of the electromagnetic tracking system is also experimentally validated.

## 1. Introduction

Pose-tracking technology is extensively employed in applications such as rehabilitation [1], virtual/augmented reality [2,3], and computer-assisted medical interventions [4] to track the movement of the human body and various tools and devices. For example, real-time pose tracking of intra-corporeal medical devices enables image registration [5], surgical navigation [6], and closed-loop robotic control [7]. Pose-tracking technology improves accuracy, safety, efficiency, and autonomy in minimally invasive medical interventions. Typical pose-tracking methods include optical tracking [8], image-based tracking [9], radio frequency (RF) positioning [10], inertial measurement unit (IMU)-based tracking [11], and magnetic tracking [4,12,13,14,15,16,17,18,19,20,21]. Optical tracking systems using infrared cameras and optical markers generally provide a high accuracy; however, optical tracking requires line-of-sight access to the target, which is not feasible in scenarios such as localization of intra-body devices in medical procedures. Image-based tracking, such as CT- and MRI-based methods, can provide intuitive guidance; however, it is not suitable for real-time tracking since it takes a long time for image acquisition and processing. RF positioning can only reach a centimeter-level accuracy, which is insufficient for many applications. IMU-based tracking offers a lightweight pose-tracking solution, which is ideal for wearable applications; however, it is susceptible to drift over time, leading to accumulated errors in position and orientation estimates.

Magnetic pose tracking is accurate and safe for the human body. Since magnetic fields can permeate most nonferrous materials, magnetic tracking does not require line-of-sight access to the target. Therefore, magnetic tracking is especially suitable for non-contact localization of intracorporeal medical devices. Magnetic pose-tracking systems are typically categorized into two types, i.e., permanent-magnet-based [14,15,16,17] and electromagnet-based [18,19,20,21] systems. Permanent-magnet-based systems utilize permanent magnets as passive magnetic markers attached to the tracked object, with external magnetic sensor arrays nearby to track these markers. Hu et al. [14] developed a permanent magnet-based 5-DoF pose-tracking system using a cubic sensor array consisting of 64 magnetic sensors. Wang et al. [15] employed the multipoint sampling method to reduce the influence of ambient disturbance on the permanent magnet-based pose-tracking system. Son et al. [16] integrated the permanent magnet-based pose-tracking system into a closed-loop electromagnetic actuation system for manipulation of untethered robots. Popek et al. [17] developed a pose-tracking system for untethered capsule robots, which uses an external rotating permanent magnet and a magnetic sensor attached to the capsule. In this system, the external permanent magnet is used for both actuation and localization of the capsule robot. There are some drawbacks of permanent magnet-based systems. First, they are susceptible to external magnetic disturbance. Furthermore, since the magnetic field of a dipole permanent magnet is rotationally symmetric about its axis of magnetic moment, this degree of freedom of orientation cannot be estimated from magnetic measurements and only 5-DoF pose tracking is feasible. In contrast, electromagnet-based pose-tracking systems are capable of 6-DoF pose tracking and are immune to external magnetic disturbance by using alternating or switched excitation of the electromagnets. Raab et al. [18] proposed the first electromagnetic pose-tracking system, which uses a three-axis transmitting coil to generate a magnetic dipole field to a three-axis receiving coil on the target. This system achieves a millimeter-level tracking accuracy but it has a large size. Yang et al. [19] developed an electromagnetic tracking system using nine transmitting coils to emit sinusoidal magnetic fields at different frequencies, which were received by a small-sized sensing coil for 5-DoF pose tracking. Anser EMT [21] was developed as an open-source electromagnetic tracking system, which integrates multiple electromagnetic coils into a flat printed circuit board. The alternating magnetic signals are received by a sensing coil for pose estimation. Most commercial magnetic tracking systems are electromagnet-based systems, such as NDI Aurora.

In magnetic tracking systems, the magnetic field near the magnetic source is modeled as a nonlinear function of the target pose. Then, the pose can be estimated from the magnetic measurements using a nonlinear estimation algorithm. Pose estimation algorithms can be categorized into analytical solutions [22,23], optimization-based methods [14,15,16,19,24], and Kalman filter-based methods [20,25,26,27,28,29]. The Kalman filter (KF)-based methods [30] are well-known for their simplicity of implementation, high computational efficiency, and high accuracy. The extended Kalman filter (EKF) broadens the application of the linear Kalman filter to nonlinear systems, such as magnetic pose-tracking systems [20]. However, Kalman filters typically treat the state space as a Euclidean space $R^{n}$, whereas the orientation state in pose-tracking systems evolves on the unit quaternion manifolds $S^{3}$. If the orientation is simply treated in the Euclidean space, the accuracy and robustness of the pose-tracking system is impaired.

A straightforward approach to addressing this issue is using minimal parameterization [31] of orientation such as Euler angles or axis–angle representation. However, both representations have inherent singularities. Unit quaternion can be used as a valid representation of orientation without the singularity issue although it is redundant. Since the standard EKF typically does not account for the unity constraint of quaternions during pose estimation, the quaternion norm errors accumulate and impair the tracking accuracy. It is noted that not only the orientation accuracy but also the position accuracy is affected because the position and orientation are coupled in the magnetic measurement model. The constrained extended Kalman filter (CEKF) [32,33], as a variant of the standard EKF, enforces algebraic constraints within the standard KF framework to avoid violations of quaternion unity. However, such constraints might cause the singularity issue in state covariance [34]. The error-state Kalman filter (ESKF) [35] operates in the local tangent space of the unit quaternion manifold, where a minimal parameterization of incremental orientation is adopted. The ESKF separates the estimated state into a nominal state and a small error state. The error state is propagated and updated using sensor measurements. After the update, the nominal state is corrected by the error state. Since the correction is conducted on the manifold, the unity of quaternion is automatically satisfied and thus the accuracy of pose estimation is enhanced. The ESKF has been widely used in inertial orientation tracking systems [36,37,38,39]; however, to the best of our knowledge, the ESKF algorithm has never been implemented in a magnetic pose-tracking system. In this paper, the ESKF is employed for pose estimation in electromagnetic tracking systems for the first time to improve the robustness and accuracy of pose tracking.

Another issue considered in this paper is the separation of magnetic fields from different electromagnetic coils and the rejection of ambient magnetic disturbance to the magnetic tracking system. Conventionally, electromagnetic tracking systems use alternating (sinusoidal) excitation of the electromagnetic coils to reject low-frequency disturbance fields, where different frequencies of excitation current are used for multiple electromagnetic coils for field separation. However, complicated demodulation algorithms and circuits are needed to separate the magnetic signals from different coils. To address this problem, a strategy of sequential coil excitation is proposed to separate the fields from different coils, which does not require complicated demodulation. In addition, the ambient disturbance field can be easily canceled by dynamically measuring the difference between the coil magnetic field and the background field.

The objective of this article is to propose and evaluate a robust tri-electromagnet-based 6-DoF pose-tracking system using an error-state Kalman filter. The system consists of three electromagnetic coils that are sequentially excited to generate dipole fields. A single three-axis magnetic sensor is attached to the target object to measure the magnetic field. A sequential excitation strategy is used to separate the magnetic fields from three coils and eliminate the external magnetic disturbance. The ESKF algorithm is utilized to achieve accurate and robust pose estimation. The major contributions of this article are listed as follows:

- The error-state Kalman filter algorithm is developed, implemented, and evaluated for the electromagnetic pose-tracking system for the first time;
- The error-state Kalman filter algorithm is compared with the conventional EKF and CEKF in terms of pose tracking accuracy and robustness using simulation and experiments;
- A strategy of sequential coil excitation is proposed to separate the magnetic measurements from different coils and reject the external magnetic disturbance.

The article is organized as follows. In Section 2.1, the working principle of the pose-tracking system is presented and the hardware and control strategy of sequential coil excitation are shown. Section 2.2 describes the pose estimation algorithms, including the standard EKF, the constrained EKF, and the proposed ESKF. Section 3 presents the simulation results of pose tracking to compare the performance of the algorithms under highly dynamic conditions. Section 4 provides experimental results of pose tracking along different trajectories to evaluate the proposed algorithm. Section 4 also demonstrates the results of magnetic disturbance rejection. The paper is concluded in Section 5.

## 2. Methodology

**Figure 1:**
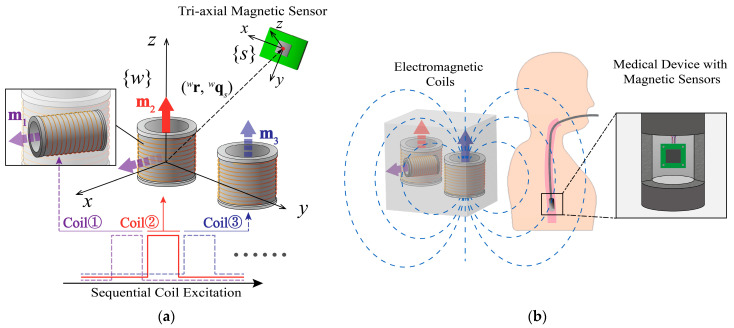
(**a**) Schematic diagram of the tri-electromagnet-based pose-tracking system (the solid lines represent currently active coils and dashed lines indicate coils queued for excitation); (**b**) the electromagnetic pose-tracking system used in a medical application of endoscope tracking.

The proposed 6-DoF pose-tracking system consists of three stationary electromagnetic coils and a tri-axial magnetic sensor attached to the moving target, as illustrated in Figure 1a. The configuration of the magnetic sources is designed with coil 1 and coil 2 being orthogonal and coil 2 and coil 3 being parallel. The three coils are excited sequentially and the magnetic sensor is used to measure the magnetic field from each coil for pose estimation. The sequential excitation of electromagnetic coils enables separation of magnetic fields from different coils and makes the pose-tracking system robust to magnetic disturbances. Once the magnetic field measurements from all three coils are collected, the 6-DoF pose of the sensor (target) can be estimated using an error-state Kalman filter, which can maintain quaternion constraints through minimal parameterization and thus enhance the accuracy and stability of pose estimation.

Figure 1b illustrates the proposed pose-tracking system in a medical application, i.e., endoscope tracking. In this example, a magnetic sensor is embedded in the endoscope to measure the magnetic fields from the three coils. The system continuously tracks the position and orientation of the endoscope, which provides accurate and intuitive guidance for the operator in medical procedures.

In this section, the magnetic measurement model is shown first, which describes the relationship between the magnetic measurements and the 6-DoF pose of the sensor. Then, the strategy of sequential coil excitation is shown to explain the mechanism of magnetic field separation and disturbance rejection. Finally, the pose estimation algorithm using an error-state Kalman filter is described.

### 2.1. Magnetic Measurement Model

A uniaxial electromagnetic coil is commonly modeled as a magnetic dipole, whose magnetic field is given by [40], as detailed below:

(1) Bew=μ04π 3mew⋅rwrw−mewrw2rw5

where $\mu_{0}=4\pi \times 10^{-7}\;T\cdot m/A$ is the magnetic permeability in a vacuum; mew denotes the magnetic moment; rw represents the relative position of the concerned point (e.g., the location of the sensor) with respect to the center of the coil; $\left\|\cdot \right\|$ represents the Euclidean norm of a vector; and Bew is the magnetic flux intensity at point rw. The left superscript indicates the reference frame in which the vector is represented. Specifically, the superscript $\left\{w\right\}$ represents the world reference frame, and $\left\{s\right\}$ represents the sensor reference frame, as depicted in Figure 1a. The relative position of the sensor to the *i*-th coil riw can be represented by the difference between the absolute position of sensor pw and the absolute position of the coil rwe,i, i.e., riw=p−rwe,iw. Thus, for the *i*-th coil, Equation (1) evolves to the following:

(2) Be,iw=μ04π 3mew⋅p−rwe,iwp−rwe,iw−mewp−rwe,iw2p−rwe,iw5

The magnetic field measurement by the sensor Be,is is obtained by rotating the magnetic field Be,iw into the sensor frame $\left\{s\right\}$.

(3) Be,is=RTswμ04π 3mew⋅p−rwe,iwp−rwe,iw−mewp−rwe,iw2p−rwe,iw5

where the Rsw is a 3 × 3 rotation matrix transforming vectors from the sensor frame to the world frame, which can represent the orientation of the sensor. To conveniently include the 3-DoF orientation into the state vector, the unit quaternion qsw=qsqvTT=qsqxqyqzT is used to represent the orientation in pose estimation. The transformation between the rotation matrix and the unit quaternion is given by the following:

(4) Rswqsw=qs2+qx2−qy2−qz22qxqy−2qsqz2qxqz+2qsqy2qxqy+2qsqzqs2−qx2+qy2−qz22qyqz−2qsqx2qxqz−2qsqy2qyqz+2qsqxqs2−qx2−qy2+qz2.

It is worth noting that magnetic measurement Be,is for each coil depends on both the position pw and the orientation qsw of the sensor. By stacking the magnetic measurements from all three electromagnetic coils, the magnetic measurement model of the tracking system is given below:

(5) y=Be,1sBe,2sBe,3s=hepw,qsw.

### 2.2. Sequential Coil Excitation for Field Separation and Disturbance Rejection

#### 2.2.1. Working Principle of Sequential Coil Excitation

It is known that the 6-DoF pose of the sensor cannot be uniquely determined by measuring the magnetic field of a single magnetic dipole source. Therefore, three electromagnetic coils are used in our proposed tracking system. If the three coils are excited simultaneously, their magnetic fields are superposed and mixed. However, to utilize the dipole-based magnetic measurement model (5) for pose estimation, the magnetic measurements of three coils should be separated. There are two methods of field separation, i.e., time-division multiplexing (TDM) and frequency-division multiplexing (FDM). In the TDM method, multiple coils are sequentially excited and their magnetic signals are independently collected. In the FDM method, multiple coils are simultaneously excited but at different frequencies. The mixed magnetic signals are then demodulated using band-pass filters to separate the signals from different magnetic sources. Since the FDM method requires complicated signal processing, the TDM method (sequential excitation) is used to separate the magnetic measurements from different electromagnets.

**Figure 2:**
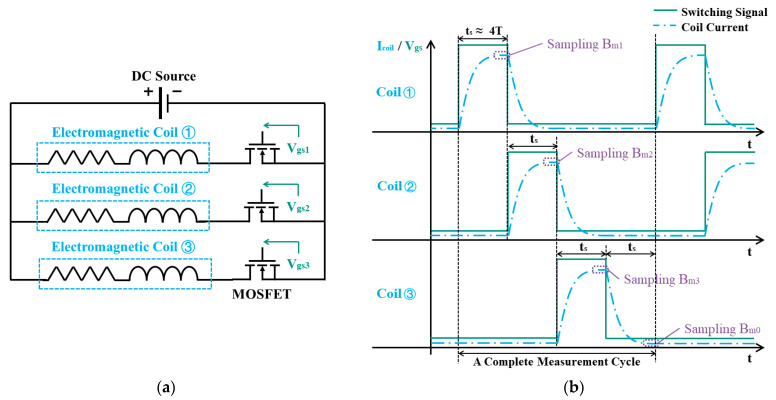
(**a**) Diagram of driving circuit for sequential coil excitation; (**b**) sequence diagram of coil excitation and the corresponding current response.

Figure 2a illustrates the circuit diagram for electromagnetic coil control. Each coil has an individual electronic switch (MOSFET, AOD4185 Alpha and Omega Semiconductor Ltd., Sunnyvale, CA, USA). When the control signal $V_{gs}$ is set high, the electronic switch is turned on and the corresponding coil is activated. The electromagnetic coil is modeled as an inductor and resistor in serial connection as shown in Figure 2a. Consequently, as illustrated in Figure 2b, when the coil is activated, the current through the coil exhibits a first-order response to the step voltage input. This dynamic response is described by the transfer function:

(6) $$I\left(s\right)=\frac{K_{e}}{Ts+1}V\left(s\right)$$

where $I\left(s\right)$ and $V\left(s\right)$ are coil current and voltage in the complex frequency domain; $K_{e}$ is the constant gain; and T is the time constant of the first-order system. Since the magnitude of magnetic moment mew of the coil is proportional to the current I, the magnetic field also experiences a first-order process before it settles down. A microcontroller is used to control the excitation of coils, including sending switching signals to activate the coil sequentially and taking stable measurements with an appropriate delay. After activating the coil, the microcontroller delays for a period of time $t_{s}$ larger than 4T to take measurements of the magnetic field, where 4T is the time required for the current response to settle down (reach approximately 98.2% of the final value). This delay ensures the successful measurement of a steady magnetic field. The time constant for an electromagnetic coil is given by $T=\frac{L}{R}$, where L is the inductance and R is the resistance of the coil. Table 1 shows the electric parameters of coils in the experiment from an LCR meter (UT622A, Uni-Trend Technology Corporation, Ltd., Dongguan, China) and the corresponding theoretical time constant of the three coils. The electromagnetic coils are designed to have a small T (<2 ms) and, thus, a small delay time $t_{s}$ (<8 ms).

As illustrated in Figure 2b, the three coils are activated sequentially. When the previous coil is switched off and enters its falling response, the next coil is immediately switched on and its current begins to rise. After a delay of $t_{s}$, the current of the previous coil drops to zero while the current of the activated coil reaches a steady state. The delay time $t_{s}$ is set to be 8 ms to accommodate the coil with the largest time constant, which ensures a stable sampling of the magnetic field from only the activated coil. At the end of a measurement cycle, all coils are deactivated to allow measurement of the ambient background magnetic field for disturbance cancellation. In each update step of pose estimation, it takes approximately 30 ms in a full measurement cycle to sample the magnetic field from three coils and the background magnetic field. This update time of magnetic field sampling is sufficient for real-time and accurate pose tracking.

#### 2.2.2. Field Separation and Disturbance Rejection

When the *i*-th electromagnetic coil is excited, the magnetic sensor measures the magnetic field from the *i*-th electromagnetic coil along with the background field, as given by the following:

(7) Bm,is=Be,is+Bbs

where Bbs is the background field, primarily consisting of geomagnetic fields and potential disturbance fields. When all three coils are deactivated, the magnetic sensor measures only the background magnetic field.

(8) Bm,0s=Bbs.

By taking the difference between the activated measurement Bm,is and the deactivated measurement Bm,0s in each measurement cycle, the separated magnetic field from each coil with no background field is acquired as follows:

(9) Be,is=Bm,is−Bm,0s.

The separated magnetic fields of electromagnetic coils are then used as the effective magnetic measurements, shown in Equation (5), for pose estimation.

The sequential coil excitation strategy also contributes to disturbance rejection. The potential disturbance field in the background field is also canceled when taking the difference between the activated and deactivated measurements as shown in Equation (9). It is assumed that the environmental magnetic disturbances remain unchanged in a complete measurement cycle. This assumption holds true as long as the period of the measurement cycle is sufficiently short.

### 2.3. Error-State Kalman Filter for Pose Estimation

The Kalman filter (KF) is a well-known optimal state estimator widely used in various applications. The extended Kalman filter (EKF) broadens the linear KF by linearizing the system kinematics and measurement models around the current state estimate using a first-order Talyor expansion, enabling accurate estimation in nonlinear systems. However, the standard EKF treats the state space as a Euclidean space $R^{n}$, which is not appropriate for the pose estimation problem where the orientation is represented using quaternions on the unit quaternions group $S^{3}$. Specifically, the unit quaternion has only three degrees of freedom, whereas the standard EKF treats it as a vector in $R^{4}$ with a missing constraint. The constrained EKF (CEKF) was proposed to mitigate this problem by enforcing unity constraints on the quaternion, forcing the redundant parameterized states back to the manifold. However, enforcing constraints might cause singularities on state covariance matrices, which impairs the stability of the system.

**Figure 3:**
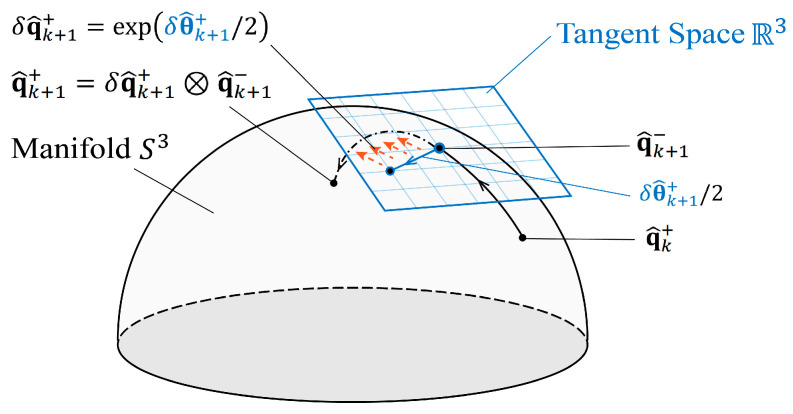
The process of quaternion estimation using ESKF. Measurement update on nearest tangent space of quaternion manifold ensures quaternion unity.

As shown in Figure 3, the error-state Kalman Filter (ESKF) operates the system on its equivalent minimally parameterized error space (i.e., the local tangent space) of the manifold, which thereby avoids singularities and redundancy in state variables and enhances the accuracy and stability of the pose-tracking system. The ESKF has been previously applied in inertial pose estimation but has never been used in magnetic pose estimation. In this paper, the ESKF is employed in the proposed 6-DoF electromagnetic pose-tracking system to improve the performance of orientation estimation. The performance of ESKF is also compared with the prevalent pose estimation algorithms of standard EKF and CEKF. In this section, all these estimation algorithms are briefly introduced.

#### 2.3.1. Standard EKF

The state vector x to be estimated consists of a 3-DoF position, translational velocity vw=vxvyvzT, and unit quaternion qsw∈S3 representing a 3-DoF orientation, which is outlined below:

(10) x=pwvwqsw.

In pose-tracking applications, the sensor can be put on any freely moving object whose kinematic model might be unknown. Thus, a trivial constant-velocity and constant-orientation kinematic model is used to describe the motion of the object, as given by the following:

(11) p˙w=vwv˙w=awq˙sw=12qsw⊗ωs

where aw=axayazT is assumed to be a zero-mean Gaussian noise that represents the unmodeled acceleration. ωs=ωxωyωzT is also assumed to be a zero-mean Gaussian noise, representing the unmodeled angular velocity. Here, ⊗ denotes the quaternion product, which can also be represented by a matrix product as follows:

(12) q˙sw=12qsw⊗ωs=12qswLωs

where qswL is the left-quaternion-product matrix, detailed below:

(13) qswL=12−qx−qy−qzqs−qzqyqzqs−qx−qyqxqs

Discretizing the system kinematic model yields the following:

(14) pk+1w=pkw+Δtvkw+12Δt2awkvk+1w=vkw+Δtawkqk+1sw=12ΔtqkswLωks

where Δt is the time step, which is equal to the sampling time of a complete measurement cycle. The discrete system process model (14) can be reformulated into a compact matrix form as follows:

(15) xk+1=Axk+GkwkA=I3ΔtI303×303×3I303×303×303×3I3Gk=12Δt2I303×3ΔtI303×304×312ΔtqkswL

where $I_{n}$ represents n×n identity matrices; and $0_{m\times n}$ denotes null matrices with the dimension of m×n. wk=awkTωkTsT is the concatenation of the process noises awk and ωks, which is modeled as a zero-mean Gaussian noise, outlined below:

(16) $$w_{k}\sim N\left(0,Q\right)$$

where the notion $N\left(\mu ,\Sigma \right)$ represents a Gaussian distribution with its mean μ and covariance Σ. The covariance matrix of the process noise $Q_{k}$ is defined by the following:

(17) $$Q_{k}=E\left(w_{k}w_{k}^{T}\right)$$

where $E\left(\cdot \right)$ is the expectation of the random variable. The process noise covariance Q is a tuning parameter that can be adjusted to achieve the desired performance of estimation.

The measurement model is simply given by Equation (5) with additive measurement noise.

(18) $$y_{k}=h_{e}\left(x_{k}\right)+B_{n,k}$$

where $B_{n,k}$ is the measurement noise vector, which is also assumed to be zero-mean and Gaussian.

(19) $$B_{n,k}\sim N\left(0,R_{k}\right)$$

where the covariance matrix of measurement noise $R_{k}$ is defined as follows:

(20) $$R_{k}=E\left(B_{n,k}B_{n,k}^{T}\right)$$

which is set according to the sensor noise in the experiment. The Jacobian of the measurement model $H_{k}$ is given by the following:

(21) Hk=∂hexk∂xx=x^k−=HpHvHqHp=Rswqsw∂Be,1w∂pwRswqsw∂Be,2w∂pwRswqsw∂Be,3w∂pwHv=09×3Hq=2qsBe,1w−qv×Be,1wqvTBe,1wI3+qvBe,1Tw−Be,1qvTw+qsBe,1w×qsBe,2w−qv×Be,2wqvTBe,2wI3+qvBe,2Tw−Be,2qvTw+qsBe,2w×qsBe,3w−qv×Be,3wqvTBe,3wI3+qvBe,3Tw−Be,3qvTw+qsBe,3w×

where $H_{p}$, $H_{v}$, and $H_{q}$ are sub-Jacobian matrices corresponding to position, velocity, and quaternion components, respectively; and ${\left[\cdot \right]}_{\times }$ represents the skew-symmetric matrix.

With the kinematic model, measurement model, and stochastic noise defined above, the standard EKF can be implemented for pose estimation [30]:

(22) $$\begin{array}{cc} {\hat{x}}_{k+1}^{-} & =A{\hat{x}}_{k}^{+}\\ P_{k+1}^{-} & =AP_{k}^{+}A^{T}+G_{k}Q_{k}G_{k}^{T}\\ K_{k+1} & =P_{k+1}^{-}H_{k+1}^{T}{\left(H_{k+1}P_{k+1}^{-}H_{k+1}^{T}+R_{k}\right)}^{-1}\\ {\hat{x}}_{k+1}^{+} & ={\hat{x}}_{k+1}^{-}+K_{k+1}\left[y_{k+1}-h_{e}\left({\hat{x}}_{k+1}^{-}\right)\right]\\ P_{k+1}^{+} & =\left(I_{10}-K_{k+1}H_{k+1}\right)P_{k+1}^{-} \end{array}$$

The standard EKF provides a basic framework for electromagnetic pose estimation; however, the problem of quaternion constraint violation caused by redundant parameterization remains unresolved for the standard EKF.

#### 2.3.2. Constrained EKF

The EKF-based estimation algorithms that enforce the unity constraint of quaternion are called constrained EKF [30]. The unity constraint of quaternion is given as follows:

(23) $$q_{s}^{2}+q_{x}^{2}+q_{y}^{2}+q_{z}^{2}=1.$$

There are many ways of enforcing the constraint while we employ the perfect measurement method [32] to enforce the unity constraint of quaternion on the basis of the standard EKF framework. This method treats the unity constraint as a measurement with zero noise.

(24) 1=hcx=qsw2

where $h_{c}(\cdot )$ is the constraint measurement function. The corresponding Jacobian of the constraint measurement model is given by the following:

(25) $$D_{k}={\left.\frac{\partial h_{c}\left(x_{k}\right)}{\partial x}\right|}_{x={\hat{x}}_{k}^{-}}={\left[\begin{array}{cc} 0_{1\times 6} & \begin{array}{cc} \begin{array}{cc} 2q_{s} & 2q_{x} \end{array} & \begin{array}{cc} 2q_{y} & 2q_{z} \end{array} \end{array} \end{array}\right]}^{T}$$

Similar to the measurement update step in the EKF framework (22), the constraint “measurement” is included in the algorithm as an additional correction step.

(26) $$\begin{array}{cc} {\tilde{K}}_{k+1} & =P_{k+1}^{+}D_{k+1}^{T}{\left(D_{k+1}P_{k+1}^{+}D_{k+1}^{T}\right)}^{-1}\\ {\tilde{x}}_{k+1}^{+} & ={\hat{x}}_{k+1}^{+}+{\tilde{K}}_{k+1}\left[1-h_{c}\left({\hat{x}}_{k+1}^{+}\right)\right]\\ {\tilde{P}}_{k+1}^{+} & =\left(I_{10}-{\tilde{K}}_{k+1}D_{k+1}\right)P_{k+1}^{+} \end{array}$$

where the variables with ”~” represent the constrained variables. This approach adds constraint measurement to project the quaternion onto the unit sphere, which maintains the validity of the orientation representation throughout the estimation process. However, this method cannot ensure that the constraint is satisfied perfectly. Additionally, a singular state covariance might appear and cause numerical problems such as ill-conditioning in the state covariance estimation [35].

#### 2.3.3. ESKF

The Error-State Kalman Filter addresses the limitations of the standard EKF and constrained EKF by utilizing an error-state formulation. This approach ensures minimal parameterization of the incremental orientation update, thereby eliminating the need for extra constraints to maintain a valid quaternion orientation representation. The core idea of the ESKF is to operate with the minimal parameterized error state δx in parallel with the nominal state x as defined in (10). The nominal state is corrected by the error state to obtain an accurate estimate of the true state.

The error-state vector δx captures small deviations from the nominal state, including position error δp, velocity error δv, and the orientation error represented by angle vector $\delta \theta \in R^{3}$.

(27) $$\delta x=\left[\begin{array}{c} \delta p\\ \delta v\\ \delta \theta \end{array}\right]$$

where the quaternion error is related with the angle error by $\delta q=\exp\left(\delta \theta /2\right)$. It is noted that the dimension of δθ is three so it is the minimal parametrization of the incremental orientation with no redundancy.

In the ESKF, the kinematic model of the nominal state is assumed to have zero noise, as given by (28).

(28) p˙w=vwv˙w=0q˙sw=0

The process noise is considered in the kinematic model of the error state.

(29) δp˙=δvδv˙=aw δθ˙=Rswωs+12δθ×Rswωs

Discretizing the error-state kinematics yields the following:

(30) δpk+1=δpk+Δtδvk+12Δt2akwδvk+1=δvk+Δtakwδθk+1=δθk+ΔtRswqswkωks+12Δtδθk×Rswqswkωks.

The discrete error-state kinematic model can be reformulated into a compact form.

(31) δxk+1=Aδxk+Gδx,kwkGδx,k=12Δt2I303×3ΔtI303×303×3ΔtRswqswk+12Δtδθk×Rswqswk

The measurement model in ESKF is the same as Equation (18), while the Jacobian of the measurement model with respect to the error state $H_{\delta x,k}$ is defined as detailed below:

(32) $$\begin{array}{cc} H_{\delta x,k} & ={\left.\frac{\partial h_{e}\left(x\right)}{\partial x}\right|}_{x={\hat{x}}_{k}^{-}}\cdot {\left.\frac{\partial x}{\partial \delta x}\right|}_{x={\hat{x}}_{k}^{-}}\\ & =H_{k}C_{k} \end{array}$$

where

(33) $$\begin{array}{cc} C_{k} & =\left[\begin{array}{ccc} I_{3} & 0_{3\times 3} & 0_{3\times 3}\\ 0_{3\times 3} & I_{3} & 0_{3\times 3}\\ 0_{4\times 3} & 0_{4\times 3} & Q_{\delta x} \end{array}\right]\\ Q_{\delta x} & =\frac{1}{2}\left[\begin{array}{ccc} \begin{array}{c} \begin{array}{c} -q_{x}\\ q_{s} \end{array}\\ \begin{array}{c} -q_{z}\\ q_{y} \end{array} \end{array} & \begin{array}{c} \begin{array}{c} -q_{y}\\ q_{z} \end{array}\\ \begin{array}{c} q_{s}\\ -q_{x} \end{array} \end{array} & \begin{array}{c} \begin{array}{c} -q_{z}\\ -q_{y} \end{array}\\ \begin{array}{c} q_{x}\\ q_{s} \end{array} \end{array} \end{array}\right] \end{array}$$

With the kinematic model and measurement model defined above, the ESKF algorithm can be implemented for pose estimation, as shown in Algorithm 1.
**Algorithm 1:** ESKF Algorithm for Electromagnetic Pose Tracking     **Input:** Magnetic measurement $y_{k+1}$, state estimate ${\hat{x}}_{k}^{+}$, and error-state covariance                 
${\tilde{P}}_{\delta x,k}^{+}$ at the previous iteration step k
    **Output:** state estimate ${\hat{x}}_{k+1}^{+}$ and state covariance ${\tilde{P}}_{\delta x,k+1}^{+}$ at the current step k+1
    **Nominal-state propagation:**        
${\hat{x}}_{k+1}^{-}=A{\hat{x}}_{k}^{+}$    
**Error-state propagation:**        
$\begin{array}{cc} {\delta \hat{x}}_{k+1}^{-} & =A\delta {\hat{x}}_{k}^{+}\\ P_{\delta x,k+1}^{-} & =A{\tilde{P}}_{\delta x,k}^{+}A^{T}+G_{\delta x,k}Q_{k}G_{\delta x,k}^{T} \end{array}$
    
**Error-state measurement update:**        
$\begin{array}{cc} K_{\delta x,k+1} & =P_{\delta x,k+1}^{-}H_{\delta x,k+1}^{T}{\left(H_{\delta x,k+1}P_{\delta x,k+1}^{-}H_{\delta x,k+1}^{T}+R_{k}\right)}^{-1}\\ {\delta \hat{x}}_{k+1}^{+} & ={\delta \hat{x}}_{k+1}^{-}+K_{\delta x,k+1}\left[y_{k+1}-h_{e}\left({\hat{x}}_{k+1}^{-}\right)\right]\\ P_{\delta x,k+1}^{+} & =\left(I_{9}-K_{\delta x,k+1}H_{\delta x,k+1}\right)P_{\delta x,k+1}^{-} \end{array}$
    
**Nominal-state correction:**        
p^wk+1+=p^wk+1−+δp^k+1+v^wk+1+=v^wk+1−+δv^k+1+q^swk+1+=expδθ^k+1+/2⊗q^swk+1−
    
**Error-state reset:**        
$\begin{array}{cc} {\delta \hat{x}}_{k+1}^{+} & =0\\ {\tilde{P}}_{\delta x,k+1}^{+} & =U_{k+1}P_{\delta x,k+1}^{+}U_{k+1}^{T} \end{array}$


**Figure 4:**
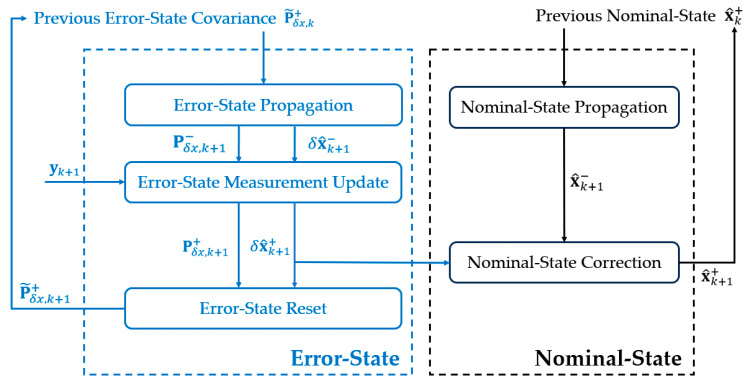
Flowchart of the ESKF algorithm for electromagnetic pose tracking.

Figure 4 provides a flowchart of major steps in the ESKF algorithm. It is seen that the nominal state is propagated according to the kinematic model (28). The propagation of the error state is trivial since the error-state estimate is reset to zero after correcting the nominal state and it remains zero in the propagation step. The error state, instead of the nominal state, is updated using the magnetic measurements. Once the error state is obtained, it is added to the nominal state to obtain the final pose estimate.

It is noted that the addition of orientation is implemented in the unit quaternions group $S^{3}$, which obviously preserves the unity of the corrected quaternion. In addition, the covariance of the error state is propagated and updated. There is no need to propagate and update the covariance of the nominal state. It is noted that the error-state covariance needs to be reset by (34) at the end of each estimation step.

(34) $${\tilde{P}}_{\delta x,k+1}^{+}=U_{k+1}P_{\delta x,k+1}^{+}U_{k+1}^{T}$$

where

(35) $$U_{k+1}=\left[\begin{array}{ccc} I_{3} & 0_{3\times 3} & 0_{3\times 3}\\ 0_{3\times 3} & I_{3} & 0_{3\times 3}\\ 0_{3\times 3} & 0_{3\times 3} & I_{3}+{\left[\delta {\hat{\theta }}_{k+1}^{+}/2\right]}_{\times } \end{array}\right].$$

In summary, the ESKF offers an elegant solution to the challenges encountered in quaternion-based pose estimation. Figure 3 demonstrates the process of quaternion estimation using the ESKF, where the measurement update steps are conducted on the tangent space and subsequently mapped back to the manifold. By utilizing a minimal parameterization of the incremental orientation in measurement update steps, i.e., small angle vector δθ, the ESKF maintains the quaternion unity without the need to enforce additional constraints. This ensures accurate orientation estimation in the electromagnetic pose-tracking system. Furthermore, ESKF reduces the risk of singularity in the state covariance caused by redundant parameterization and, thus, enhances the stability of the pose estimation system.

## 3. Simulation Results

A simulation is conducted to evaluate the performance of the proposed ESKF algorithm in electromagnetic pose estimation, particularly under the conditions where the rotation of the sensor is rapid. The ESKF is also compared with the standard EKF and CEKF. The simulation of the fast-rotation scenario is used to highlight the difference between the three estimation algorithms and reveal the advantage of the proposed ESKF algorithm.

The magnetic moments and center positions of three electromagnetic coils used in the simulation are given below.

m1w=02.50TA⋅m2rwe,1=000Tmm2w=002.5TA⋅m2rwe,2=000Tmm3w=002.5TA⋅m2rwe,3=−0.0500Tm

**Figure 5:**
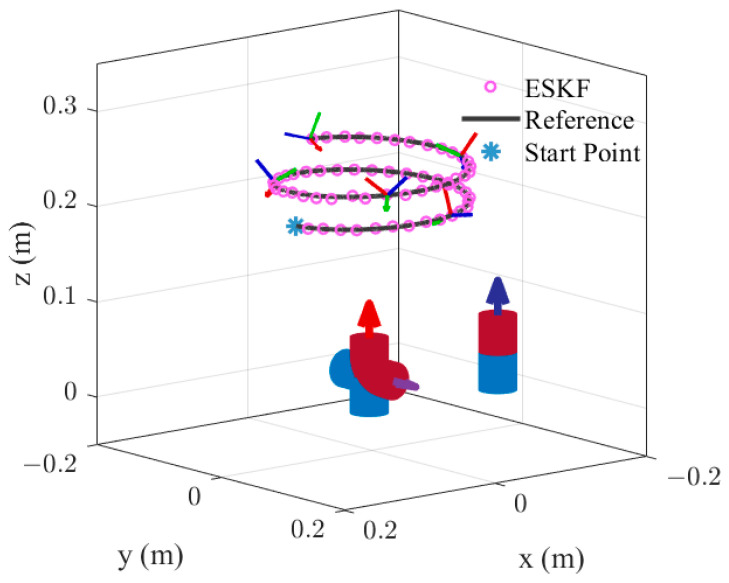
Simulation results of pose estimation.

The sensor noise in the simulation is set to be zero-mean, white, and Gaussian with a standard deviation of $10^{-7}T$. As shown in Figure 5, the sensor moves along a helical trajectory. For translation, the trajectory started from position $\left[\begin{array}{ccc} 100 & 0 & 2 \end{array}00\right]mm$, the velocity in *z*-axis is 0.001 m/s, the projection of the trajectory on the XOY-plane is a circular trajectory with a radius of 0.1 m and an angular rate of 0.0018 rad/s. The rotational speed of the sensor is ωst=0.1050.1050.105Trad/s throughout the trajectory. The initial point in the algorithms is set close to the ground truth. The entire simulation lasts for 60 s with an update rate of 100 Hz. The covariance for process and measurement noise of all three estimation algorithms are set as follows:

(36) $$\begin{array}{cc} Q_{k} & =\left[\begin{array}{cc} 164\;I_{3}{\left(m/s^{2}\right)}^{2} & 0_{3\times 3}\\ 0_{3\times 3} & 92\;I_{3}{\left(rad/s\right)}^{2} \end{array}\right]\\ R_{k} & =10^{-14}I_{9}{\left(T\right)}^{2} \end{array}$$

**Figure 6:**
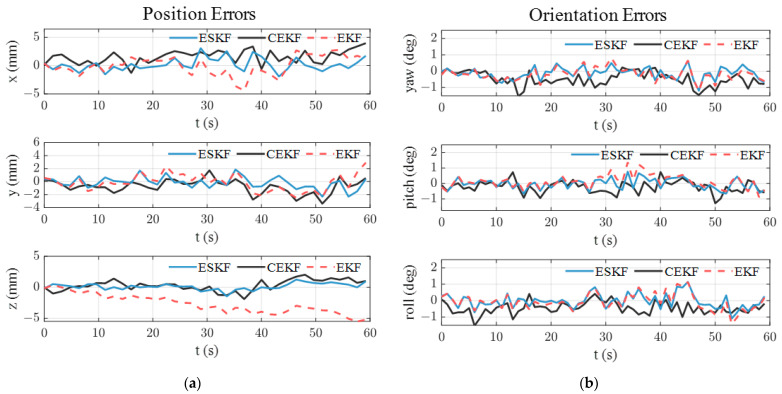
Pose estimation errors in simulation: (**a**) position errors; (**b**) orientation errors.

Figure 6 illustrates the pose tracking error over time in the simulation. In Figure 6a, the standard EKF exhibits the largest position tracking error and shows a drift beyond 5 mm in the *z*-axis. As shown in Table 2, the ESKF has the smallest position and orientation error. Specifically, the Euclidean position error of ESKF is 1.74 mm and the average Euler angle error of the ESKF is 0.42°. It is noted that the proposed ESKF shows a significantly smaller position error while it has a limited advantage in orientation tracking.

**Figure 7:**
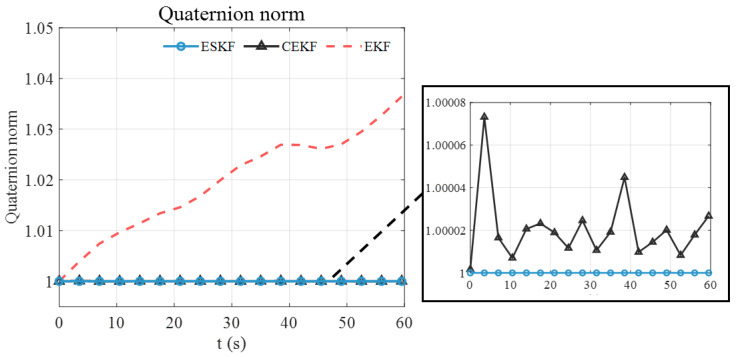
Norm of the estimated quaternion using standard EKF, CEKF, and ESKF in simulation.

To explain the advantage of the ESKF in pose tracking, the norm of the estimated quaternion using the standard EKF, CEKF, and the ESKF is analyzed. As illustrated in Figure 7, the norm of the estimated quaternion using standard EKF keeps increasing, deviating from unity continuously and reaching up to 1.036. It is known that the position and orientation are coupled in the electromagnetic pose-tracking system (see the measurement models (3) and (5)). Therefore, the violation of the quaternion constraint means that the determinant of rotation matrix Rswqsw also deviates from unity, thereby distorting the translation part in measurement model (3) and causing large position errors. The EKF shows an increasing position error in Figure 6a because of the growing violation of the quaternion constraint. The subfigure of Figure 7 shows the norm of the estimated quaternion using the CEKF and ESKF. The maximum norm error of the CEKF is $7.32\times 10^{-5}$ and that of ESKF is only 5$.32\times 10^{-15}$, which is close to the magnitude of machine precision in MATLAB ($\epsilon =2.22\times 10^{-16}$). Hence, the ESKF has the smallest position tracking error.

In summary, the proposed electromagnetic pose-tracking system using ESKF demonstrated a significantly superior position accuracy and a slightly better orientation accuracy by maintaining the quaternion unity.

## 4. Experimental Results

### 4.1. Experimental Setup

**Figure 8:**
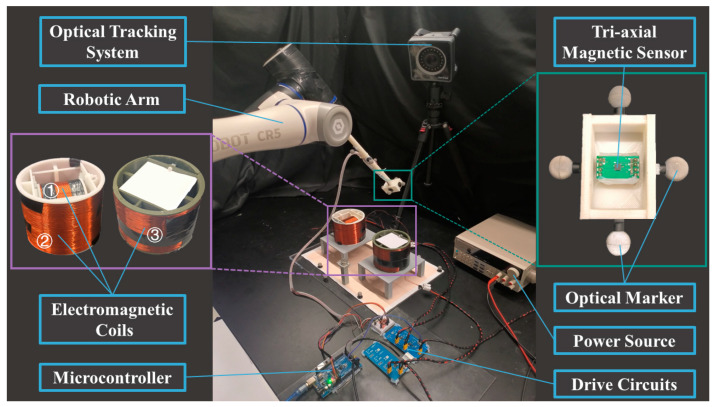
Experimental setup for evaluation of electromagnetic pose-tracking system.

Figure 8 shows the experimental setup for evaluation of the 6-DoF tri-electromagnet pose-tracking system. The center positions and magnetic moments of the three electromagnetic coils used in the experiment are given below.

m1w=02.080.02TA⋅m2rwe,1=000.10Tmm2w=0.420.22−6.09TA⋅m2rwe,2=000.10Tmm3w=0.45−0.16−4.68TA⋅m2rwe,3=−0.19−0.010.10Tm

Coil 1 and coil 2 are collocated and orthogonal and coil 2 and coil 3 are parallel. Coil 2 and coil 3 have the same size of ∅100 mm×87 mm, while the size of coil 1 is ∅64 mm×50 mm. The electric parameters of the coils can be found in Table 1. The electromagnetic coils are controlled by the driving circuits outlined in Section 2.2, running at a sampling rate of 30 Hz for a complete measurement cycle.

As illustrated in the subfigure of Figure 8, a tri-axial anisotropic magnetoresistive (AMR) magnetic sensor (MMC5983MA, MEMSIC Semiconductor Co., Ltd., Tianjin, China) was used with on-chip signal processing and integrated I2C bus. The sensor has a scale range of ±8 Gauss with an integrated 18-bit ADC and a noise level of 0.4 mGauss. A microcontroller (ATmega2560, Microchip Technology Inc., Chandler, AZ, USA) was employed to collect data from the sensor through the I2C bus and to control the coils. The sensor data were transmitted to a personal computer for implementing the pose estimation algorithm.

A robotic arm (CR5, DOBOT Robotics Co., Ltd., Shenzhen, China) was used to move the sensor along the testing trajectories. An optical tracking system (PrimeX 22, NaturalPoint Inc., Corvallis, OR, USA) was used to provide the reference pose of the sensor with a sub-millimeter and sub-degree accuracy.

### 4.2. Pose Estimation Results

In this section, the pose estimation experiments are conducted along two testing trajectories to evaluate the performance of the electromagnetic pose-tracking system. The accuracy and robustness of the proposed ESKF pose estimation algorithm are evaluated for different dynamics of motion. The covariance of process and measurement noise are kept the same for all three algorithms, which are given by the following:

(37) $$\begin{array}{cc} Q_{k} & =\left[\begin{array}{cc} 1\;I_{3}{\left(m/s^{2}\right)}^{2} & 0_{3\times 3}\\ 0_{3\times 3} & 0.64\;I_{3}{\left(rad/s\right)}^{2} \end{array}\right]\\ R_{k} & =10^{-14}I_{9}{\left(T\right)}^{2} \end{array}$$

**Figure 9:**
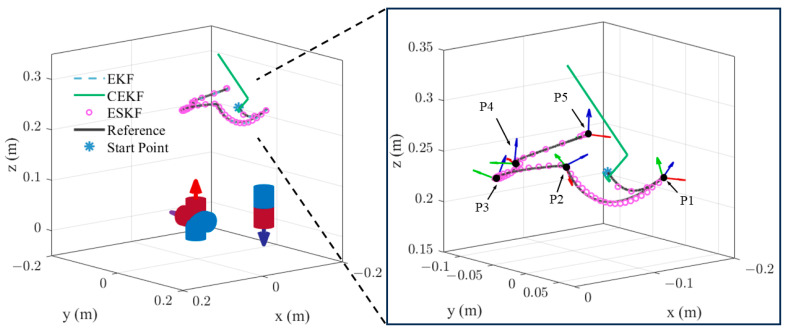
Experimental pose tracking results for the first testing trajectory.

The first testing trajectory, as illustrated in Figure 9, consists of four arcs and a linear segment. The waypoints of the first testing trajectory are pointed in the subfigure of Figure 9, with their coordinates and orientations listed in Table 3. As the sensor moves along the trajectory, it rapidly rotates as shown in the subfigure of Figure 9, where the tri-axis frames represent the sensor reference frame along the trajectory. The initial point in the algorithms is set close to the start point P0. It is shown that both the standard EKF and ESKF track the true pose trajectory well. Nevertheless, CEKF diverges shortly after the test begins, which indicates that the CEKF is potentially unstable for certain trajectories with fast rotations.

**Figure 10:**
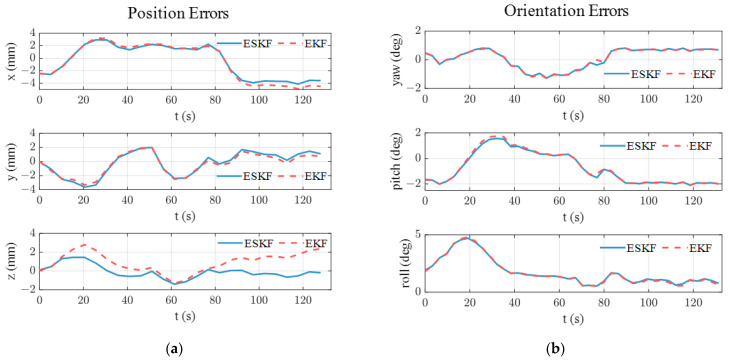
Pose estimation errors for the first testing trajectory: (**a**) position errors. (**b**) orientation errors.

The instability of the CEKF results in enormous pose tracking errors. Therefore, the tracking errors of the CEKF are excluded from Figure 10 and Table 3. As shown in Figure 10a, the standard EKF exhibits a larger position tracking error than the ESKF, particularly in the *z*-axis. Table 4 presents the RMS pose tracking error in the experiment. It is shown that the *z*-axis position error of the standard EKF is approximately two times larger than that of ESKF. The overall Euclidean error for standard EKF is 3.63 mm, which is 0.34 mm larger than ESKF. However, similar to the simulation results, the orientation errors of the two algorithms are quite close, which indicates that orientation errors caused by incorrect quaternion normalization might be overshadowed by other influential factors such as magnetic field model errors.

**Figure 11:**
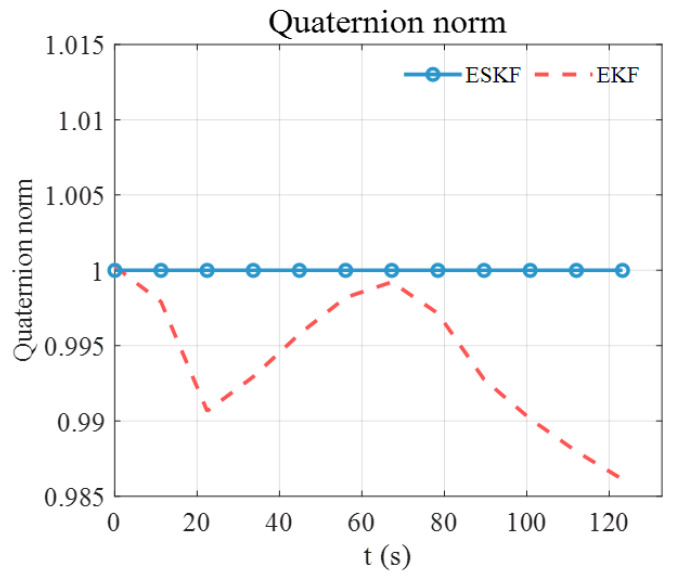
Norm of the estimated quaternion using standard EKF and ESKF for the first testing trajectory.

The norm of the estimated quaternion is illustrated in Figure 11. The norm of the quaternion estimated by the standard EKF deviates from unity significantly. Moreover, the two peaks of the deviation pattern correspond to the two peaks of the *z*-axis position error of the standard EKF in Figure 10a. This observation reinforces the argument in Section 3 that the large position error of the EKF is caused by the unnormalized quaternions. The norm of the quaternion estimated by the ESKF has the largest deviation of $3.55\times 10^{-15}$, ensuring accurate and robust pose estimation.

**Figure 12:**
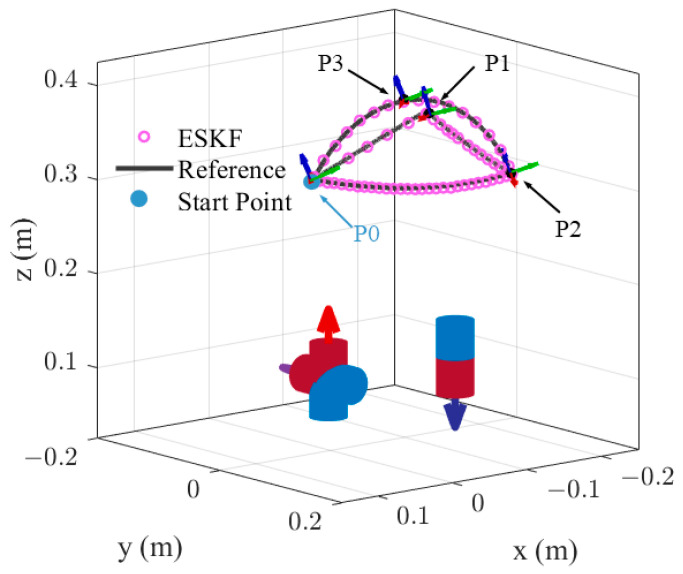
Pose tracking results for the second testing trajectory.

The second testing trajectory, as illustrated in Figure 12, is composed of a closed triangular segment followed by two repeating arcs with varying linear velocity. Table 5 lists the critical waypoints. The closed triangular trajectory consists of three segments, which are linear routes from P0 to P1, P1 to P2, and an arc from P2 back to P0. The next segment is an arc from P0 to P2 with midpoint P3, followed by an arc from P2 back to P0. The final part repeats the arc from P0 to P2 with midpoint P3 and the arc from P2 back to P0 but with a slower velocity. The rotation of the sensor is moderate compared with the first trajectory. The initial point in the algorithms is set to the same point and close to the start point P0. All three algorithms can achieve successful 6-DoF pose tracking in this test scenario with a slow and small-range rotation.

The pose tracking errors for the second testing trajectory are shown in Table 6. The orientation errors of the three algorithms range from 0.42° to 0.47°, which are approximately the same. However, the position errors of the ESKF and CEKF are smaller than that of the standard EKF. Specifically, the Euclidean position errors of the standard EKF, CEKF, and ESKF are 2.46 mm, 2.25 mm, and 2.23 mm, respectively. The experiment for the second testing trajectory demonstrates that the ESKF can consistently achieve robust and accurate pose estimation. The CEKF can achieve acceptable tracking performance under less challenging conditions, with slow and small-range rotations.

In summary, experimental results for both testing trajectories confirm that the ESKF is the most robust and accurate estimation algorithm for electromagnetic pose tracking. The standard EKF struggles with significant position errors due to the violation of the quaternion unity constraint. Although the CEKF works well in some scenarios, it shows instability and potentially diverges for certain challenging trajectories with fast and large-range rotations. These experimental findings conclude that the proposed 6-DoF tri-electromagnet pose-tracking system using the ESKF algorithm is reliable and accurate.

### 4.3. Disturbance Rejection Performance

A fundamental problem with magnetic pose-tracking systems is their susceptibility to environmental magnetic disturbances. It is claimed that the proposed system can reject magnetic disturbances by using the strategy of sequential coil excitation and background field cancellation. In this section, the disturbance rejection capability of the proposed electromagnetic pose-tracking system is experimentally validated.

**Figure 13:**
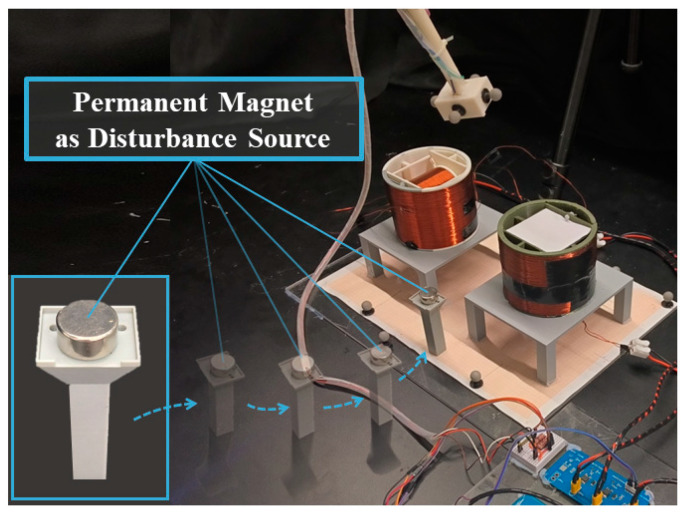
Experimental setup for validation of disturbance rejection.

The experimental setup for disturbance rejection validation is illustrated in Figure 13. During this experiment, the sensor follows a trajectory similar to the second trajectory in Section 4.2. Meanwhile, a NdFeB permanent magnet (∅20×10 mm, $m=1.8\;A\cdot m^{2}$) is manually moved toward the workspace of the tracking system and then kept near the setup, as depicted in Figure 13. The permanent magnet can exert a strong disturbance field larger than 500 mGauss.

Pose estimation experiments using the ESKF were repeated with and without the disturbance of the permanent magnet. Table 7 illustrates the RMS pose tracking errors with and without the disturbance. The Euclidean position error with disturbance is 2.21 mm, which is approximately the same as that without the disturbance (2.23 mm). The average orientation error also remains the same when the disturbance is added. Therefore, it is validated that the proposed electromagnetic pose-tracking system is robust to magnetic disturbances.

**Figure 14:**
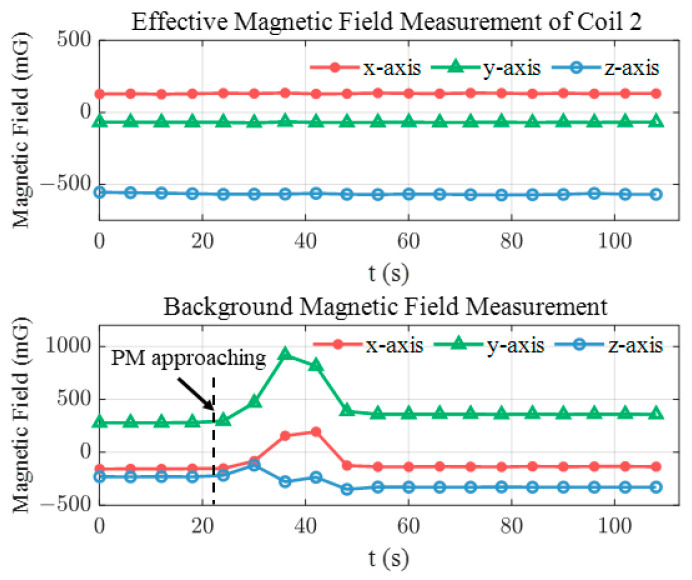
The dipole field measurement in presence of disturbance.

To reveal the underlying mechanism of disturbance rejection, the effective magnetic measurement of coil 2 Be,2s and the background magnetic field measurement Bm,0s are plotted in Figure 14. In this test, the sensor is held stationary at the center of the workspace and then the permanent magnet (PM) is introduced. When the PM approaches the tracking system, the background field measurement is influenced by the disturbance significantly while the effective magnetic measurement used for pose estimation remains unaffected due to the cancellation of the background field with disturbance, i.e., Be,2s=Bm,2s−Bm,0s.

## 5. Conclusions

In this article, a robust tri-electromagnet-based 6-DoF pose-tracking system is proposed using an error-state Kalman filter algorithm. The pose-tracking system consists of three stationary electromagnetic coils and a magnetic sensor attached to the moving target. By employing a sequential excitation strategy, the system can effectively separate the magnetic fields of three coils and cancel the environmental magnetic disturbances. The magnetic measurements from three coils are fused using an error-state Kalman filter, which fundamentally avoids the violation of quaternion unity and achieves accurate and robust pose estimation.

A simulation is designed to evaluate the proposed system under a challenging condition with rapid and large-range rotations of the sensor. Simulation results show that the RMS Euclidean position tracking error is 1.74 mm and the average orientation error is 0.42°. Although the orientation tracking error of the proposed ESKF is only slightly smaller than those using the standard EKF and CEKF, the proposed system using the ESKF shows a significant improvement in position tracking accuracy.

Experiments were conducted to validate the pose-tracking system for two trajectories with different dynamics. The experimental results demonstrate that position tracking performance is significantly improved by the ESKF compared to the traditional standard EKF, while the orientation tracking errors are almost identical. It is found that the violation of quaternion unity can significantly distort position estimation due to the coupling effect of position and orientation in the measurement model, which leads to large position tracking errors in the standard EKF. Nevertheless, the orientation estimation is only slightly affected by the quaternion normalization error, resulting in the slight advantage of ESKF in orientation estimation. While the CEKF can preserve the unity of quaternion and achieve a high accuracy close to ESKF, it shows instability in pose estimation in certain challenging dynamics of motion. Overall, the proposed ESKF stands out for its enhanced accuracy and robustness.

An experiment was conducted to validate the disturbance rejection performance of the proposed pose-tracking system. It is shown that the strategy of sequential coil excitation and background field cancellation can effectively remove magnetic disturbances. The Euclidean position tracking error is 2.21 mm and the average orientation angle error is 0.45° in presence of the disturbance from a permanent magnet.

Future works include optimizing the electromagnetic tracking system to increase the update rate and accuracy and evaluating the tracking system in applications of medical robot navigation.

## Figures and Tables

**Table 1:** Electric parameters and time constant of the coils.

	Inductance/mH	Resistance/Ω	Time Constant/ms
Coil 1	4.27	4.35	0.98
Coil 2	8.40	4.74	1.77
Coil 3	8.41	4.67	1.80

**Table 2:** RMS pose tracking errors in simulation.

		Standard EKF	CEKF	ESKF
Position Error (mm)	x	2.12	1.75	1.33
	y	1.31	1.36	0.96
	z	3.14	0.84	0.57
	Euclidean	4.02	2.37	1.74
Orientation error (°)	Yaw (ψ)	0.46	0.61	0.42
	Pitch (θ)	0.45	0.45	0.39
	Roll (ϕ)	0.49	0.60	0.45
	Average	0.47	0.55	0.42

**Table 3:** Waypoints for the first testing trajectory.

	Position (mm)	Orientation ^1^ (°)
Start Point P0	(−132, −35, 226)	(−168, 12, 0)
P1	(−130, 55, 239)	(−160, −2, 40)
P2	(−24, 34, 256)	(−150, 57, 40)
P3	(−44, −97, 221)	(125, 26, 2)
P4	(−54, −82, 238)	(112, 6, 2)
P5	(−131, −64, 259)	(−171, 15, −16)

^1^ The orientations Rswqsw are represented by Z-Y-X (yaw, pitch, roll) Euler angles.

**Table 4:** RMS pose tracking errors for the first testing trajectory.

		Standard EKF	ESKF
Position Error (mm)	x	2.97	2.71
	y	1.55	1.74
	z	1.38	0.71
	Euclidean	3.63	3.29
Orientation Error (°)	Yaw (ψ)	0.70	0.71
	Pitch (θ)	1.42	1.45
	Roll (ϕ)	2.04	2.12
	Average	1.39	1.43

**Table 5:** Waypoints for the second testing trajectory.

	Position (mm)	Orientation (°)
Start Point P0	(70, 57, 330)	(−50, −4, −35)
P1	(−70, 77, 385)	(−28, −4, −17)
P2	(−195, 57, 300)	(−60, −22, −2)
P3	(−54, 57, 400)	(−50, −12, −20)

**Table 6:** RMS pose tracking errors for the second testing trajectory.

		Standard EKF	CEKF	ESKF
Position Error (mm)	X	1.52	1.45	1.43
	Y	1.67	1.57	1.53
	Z	0.97	0.70	0.75
	Euclidean	2.46	2.25	2.23
Orientation Error (°)	Yaw (ψ)	0.39	0.39	0.38
	Pitch (θ)	0.52	0.41	0.52
	Roll (ϕ)	0.49	0.47	0.46
	Average	0.47	0.42	0.45

**Table 7:** RMS pose tracking errors with and without disturbance using ESKF.

		No Disturbance	Disturbance Added
Position Error (mm)	X	1.43	1.47
	Y	1.53	1.51
	Z	0.75	0.66
	Euclidean	2.23	2.21
Orientation Error (°)	Yaw (ψ)	0.38	0.44
	Pitch (θ)	0.52	0.49
	Roll (ϕ)	0.46	0.43
	Average	0.45	0.45

## Data Availability

All the relevant data supporting the findings of this study are available within the paper.
